# Monitoring Mosquito Abundance: Comparing an Optical Sensor with a Trapping Method

**DOI:** 10.3390/insects15080584

**Published:** 2024-08-01

**Authors:** Topu Saha, Adrien P. Genoud, Gregory M. Williams, Gareth J. Russell, Benjamin P. Thomas

**Affiliations:** 1 Department of Physics, New Jersey Institute of Technology, University Heights, Newark, NJ 07102, USA; ts627@njit.edu; 2 Centre national de la recherche scientifique, Institut Lumière Matière, Universite Claude Bernard Lyon 1, UMR5306, F-69622 Villeurbanne, France; adrien.genoud@univ-lyon1.fr; 3 Center for Vector Biology, Rutgers University, New Brunswick, NJ 08901, USA; gwilliams@hudsonregional.gov; 4 Department of Biological Sciences, New Jersey Institute of Technology, University Heights, Newark, NJ 07102, USA; russell@njit.edu

**Keywords:** mosquitoes, culicidae, insect abundance, trap, optical sensors, population monitoring, vector control

## Abstract

**Simple Summary:**

Over the last decade, optical sensors have demonstrated great potential to provide complementary data to monitor insect abundance. This article introduces a field-deployable and non-destructive optical instrument to monitor the abundance of flying insects, called an Entomological Bistatic Optical Sensor System, or eBoss. The study focuses on comparing abundance measurements made by the eBoss and physical traps over an 8-month field campaign. The eBoss made over 302,000 insect observations and evaluated the aerial density (#/m^3^) of all flying insects as well as specifically male and female mosquitoes with a 1 min resolution, allowing us to monitor both the abundance over the season and daily peak of activity. The study’s objectives were to validate the optical sensor’s data against physical trap collections, which confirmed the correlation between the two methods. However, the eBoss demonstrated superior temporal resolution (1 min versus approximately 3 days) and statistical power due to its larger sample size. These findings suggest that an eBoss can significantly enhance flying insect monitoring efforts, such as mosquitoes or pollinators, providing valuable insights for vector control strategies, agriculture and public health planning.

**Abstract:**

Optical sensors have shown significant promise in offering additional data to track insect populations. This article presents a comparative study between abundance measurements obtained from a novel near-infrared optical sensor and physical traps. The optical instrument, named an Entomological Bistatic Optical Sensor System, or eBoss, is a non-destructive sensor operating in the near-infrared spectral range and designed to continuously monitor the population of flying insects. The research compares the mosquito aerial density (#/m^3^) obtained through the eBoss with trap counts from eight physical traps during an eight-month field study. The eBoss recorded over 302,000 insect sightings and assessed the aerial density of all airborne insects as well as male and female mosquitoes specifically with a resolution of one minute. This capability allows for monitoring population trends throughout the season as well as daily activity peaks. The results affirmed the correlation between the two methods. While optical instruments do not match traps in terms of taxonomic accuracy, the eBoss offered greater temporal resolution (one minute versus roughly three days) and statistical significance owing to its much larger sample size. These outcomes further indicate that entomological optical sensors can provide valuable complementary data to more common methods to monitor flying insect populations, such as mosquitoes or pollinators.

## 1. Introduction

Entomological photonic sensors have seen important developments in recent decades, with many studies making use of entomological lidars [1,2,3,4,5,6,7,8,9,10,11,12,13,14] and various types of optical sensors [15,16,17,18,19,20,21,22,23,24,25,26,27,28,29,30]. They provide a valuable alternative to more common methods to monitor insect populations and behavior, such as baited or non-baited physical traps [19]. While optical sensors do not have the taxonomic accuracy that can be reached by physically capturing the insects, they can observe a very large number of flying insects, typically in the range of thousands per day allowing them to track insect abundance with a temporal resolution in the minute range. The data collection and processing can be conducted automatically, which allows for an uninterrupted measurement campaign throughout the season, e.g., eight consecutive months in Saha et al. [31,32], and may greatly reduce the labor cost associated with traps. Thanks to their high temporal resolution and continuous operation, the daily activity of flying insects and the dynamics of their population over the season can be measured. In addition, one can study the impact of various factors on insect populations, such as the atmospheric conditions [31] (temperature, relative humidity, sunlight exposure, wind speed, rain), the effects of pesticide applications [33], or light or noise pollution to name a few [34,35].

Over the last three years, our team at NJIT has developed an entomological optical sensor with the objective of evaluating the daily activity and population dynamics of flying insects. The instrument is an Entomological Bistatic Optical Sensor System, or eBoss, and is based on measuring the optical extinction of flying insects passing through the fixed horizontal beam of a near-infrared laser source. The system is designed for long-term campaigns; it can operate 24/7 and resists harsh weather conditions and a large range of temperatures. It requires no supervision and can observe thousands of insects per day. The data can be processed automatically, which removes the labor-intensive aspect of collecting and identifying insects associated with physical traps. Once processed, the data provide the aerial density (number of flying insects per cubic meter) and biomass density (mass of flying insects per cubic meter) [15,31]. The system is affordable (<USD 3000) and, most importantly, eye-safe. These two aspects are key to considering the future deployment of a network of sensors to achieve the monitoring of insect populations with larger spatial coverage.

The present study focuses on a field campaign from April to December 2022, with the objective of comparing the mosquito abundance retrieved by the eBoss and a network of light traps operated by the Mosquito Program of the Hudson Regional Health Commission.

## 2. Materials and Methods

### 2.1. Entomological Bistatic Optical Sensor System (eBoss)

**Figure 1:**
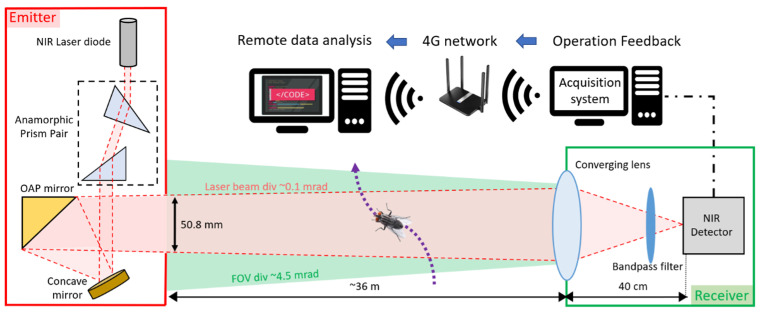
Optical layout of the eBoss instrument.

The eBoss was inspired from previous work on lidar and stand-off sensors for atmospheric studies [36,37,38,39]. It is a bistatic instrument with an emitter and a receiver positioned between 10 and 100 m from each other (36 m distance in the present campaign). As shown in Figure 1, the emitter side holds the laser source, which is a 5 mW continuous near-infrared (NIR) laser diode (CPS980, Thorlabs, Newton, NJ, USA) with a wavelength of 980 nm at 20 °C. The source emits an elliptical-shaped laser beam, transformed into a circular Gaussian beam by an anamorphic prism pair with 3× magnification. Subsequently, the beam is directed towards a concave mirror and reflected by an off-axis parabolic (OAP) gold mirror, with both mirrors acting as a beam expander. The OAP mirror reflects only the central portion of the beam with a diameter of 50.8 mm, effectively removing the wings of the Gaussian beam to ensure a uniform energy density of the laser beam throughout the entire optical path. The laser beam propagates at a height between 20 cm to a maximum of 80 cm from the ground. The beam is pointed toward the converging lens of the receiver with an effective focal length of 40 cm. The light is focused onto the active area of a silicon amplified photodetector (PDA36A2, Thorlabs, Newton, NJ, USA) after passing through a spectral bandpass filter (950–1000 nm wavelength). The optical signal is recorded at a sampling frequency of 30,517 Hz using a 16-bit digitizer (M4i4420- × 8, Spectrum, Hackensack, NJ, USA) with a 3 V range. The acquisition system is integrated into a standard desktop computer (Dell Technologies, Round Rock, TX, USA), connected via a 4G LTE router (Router: Cudy, Shenzhen, China, WiFi 6 Router AX1800, network provider: T-mobile, Bonn, Germany) for remote access to monitor the proper functioning of the system. Data are saved locally and while access to a 4G network is not necessary to operate the eBoss, it allows for the rapid detection of system failures and removes the need to check in-person the proper functioning of the system. A weather station (WS-1002-WIFI, Ambient Weather, Chandler, AZ, USA) is positioned about 15 m away from the receiver to monitor the meteorological conditions with a one-minute resolution. The eBoss was installed in a field within the city of Secaucus (Hudson County, NJ, USA); it is approximately 40 m × 10 m and bordered by a roughly 1 ha woodlot. The instrument was deployed on 20 April, and data were collected until 21 December 2022. The system was inspected once a week to monitor its proper functioning, notably to verify the power, alignment, pointing and shape of the laser beam; corrective measures were necessary approximately once per month.

#### Data Analysis

**Figure 2:**
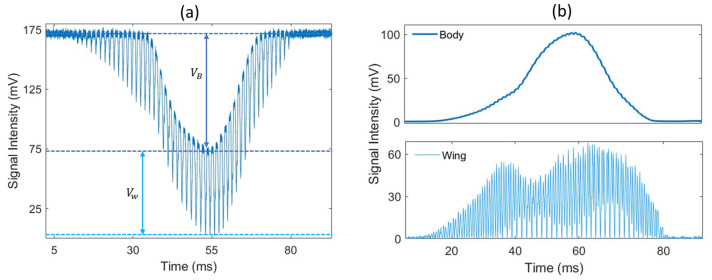
(**a**) An example of an optical signal caused by a flying insect; (**b**) change in signal intensity due to the insect’s body (**top**) and wing (**bottom**) contribution. The optical extinction cross-section of the wings and body is derived from the drop in voltage $V_{w}$ and $V_{B}$, respectively.

The measured signal corresponds to the light transmitted to the detector on the receiver side. When an insect transits through the laser beam, the insect’s body and wings scatter, diffract and absorb part of the incident light, causing the optical extinction along the optical path to significantly increase, leading to a drop in the signal from its baseline value with a somewhat Gaussian shape. This drop in signal, referred to as a transit signal, displays amplitude oscillations caused by the rapid movement of the wings. An example of such a signal is presented in Figure 2a, and the wing and body contributions are separated in Figure 2b. By detecting local minimums, the wing signal can be separated from the body signal so that the optical extinction cross-section can be retrieved for both the wings and body separately (here, body refers to everything that is not the wings). The typical duration of a transit event is in the 100 ms range. Events lasting less than 10 ms are automatically disregarded as they are too short to record enough wing movement cycles, and the longest events can approach 1 s. These events can also be triggered by objects other than insects, such as falling leaves, large pollen particles, water droplets, or any other object entering the laser beam. To identify insect events, a detection algorithm is employed to recognize drops in the signal that fall below a specific threshold. The distinctive wing patterns are then used to differentiate between insects and other non-insect objects. The details of this algorithm have been comprehensively documented previously [15]. In the case of rain, water droplets generate transit signals that can be distinguished from insect transit signals due to their lack of frequency components in the 10–900 Hz frequency range. For light rain, these events can be effectively filtered out and do not hinder the proper data collection process. However, during heavy rain, a continuous stream of water droplets passing through the beam makes it impossible to identify potential insect transits.

Through the measurement of numerous daily events, the sensor has the capability to assess the aerial density of airborne insects. Instead of a simple count of transits, this approach takes into account the transit time of each event, which is related to the insect’s flight velocity. Insects that fly quickly, like flies, are more likely to interact with the instrument as they cover more distance in a given time but have a shorter transit time. In contrast, slower insects like butterflies or moths are less likely to enter the instrument’s field of view but spend more time within the laser beam. Consequently, counting events inherently introduces a bias toward fast-flying insects, and the count value is directly linked to the instrument’s probe volume, rendering it a relative measure of abundance, as opposed to aerial density, which is an absolute metric and eliminates the bias regarding insect flight velocity. The aerial density is determined using Equation (1), where the sum of all transit times is normalized by the measurement duration and the volume surveyed by the instrument. The result is expressed in the number of insects per cubic meter of air. This methodology has been extensively discussed by Genoud et al. and validated through numerical simulations [7].

(1) $$\rho_{a}=\frac{\sum_{i}{\Delta t}_{i}}{T\cdot V}$$

where $\rho_{a}$ is the aerial density expressed in insect/m^3^, ${\Delta t}_{i}$ is the transit time of event i, and V is the probe volume of air. T is the duration during which $\rho_{a}$ is measured and defines the temporal resolution of $\rho_{a}$. This temporal resolution can be chosen in the minute range to observe insect behavior on the short time scale or in the daily or weekly range to observe long-term trends in population dynamics. Note that this temporal resolution is related to the retrieved aerial density and is distinct from the temporal resolution of the measured optical signal (≈33 μs).

**Figure 3:**
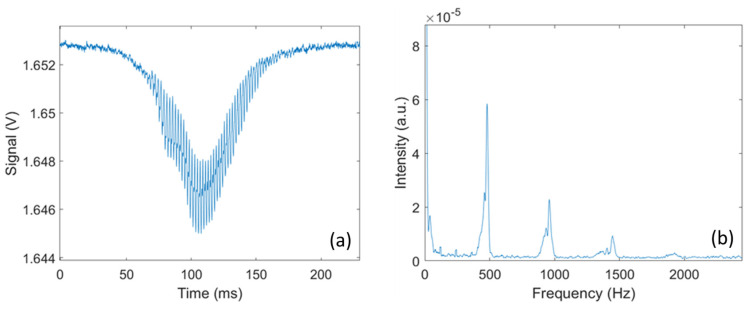
(**a**) An example of an optical signal caused by a flying mosquito; (**b**) FFT analysis on the transit signal, showing harmonic peaks with first and fundamental peaks at around 500 Hz.

Each transit signal is then analyzed to retrieve additional information on the insect responsible for the signal. As illustrated in Figure 3, by conducting a frequency analysis of the transit signal, the wingbeat frequency $f_{w}$ and its harmonics can be retrieved. The wingbeat frequency is retrieved by performing a Fast Fourier Transform on the transit signal and using a peak detection algorithm to identify the fundamental frequency of the signal. In addition, by measuring the amplitude of the body and wing signals, the optical extinction cross-section for the wings and body can be retrieved. In the absence of any target, the voltage measured by the detector is referred to as the background signal $V_{0}$, and the voltage measured during the transit is noted $V_{w}$ and $V_{B}$ for the wings and body, respectively. Knowing the cross-section area *A* of the laser beam, the optical extinction cross-section for the wings ($\sigma_{W}$) and body ($\sigma_{B}$) can be retrieved using Equations (2a) and (2b).

(2a) $$\sigma_{w}=\frac{V_{0}-V_{w}}{V_{0}}\times A$$

(2b) $$\sigma_{B}=\frac{V_{0}-V_{B}}{V_{0}}\times A$$

Both $V_{W}$ and $V_{B}$ are evaluated by taking the average of the 10% lowest values. Finally, the ratio of the wing-to-body cross-section can be evaluated using the following Equation (3).

(3) $$\sigma_{w/b}=\frac{\sigma_{w}}{\sigma_{w}+\sigma_{B}}$$

### 2.2. Trap Data Collection Method

**Figure 4:**
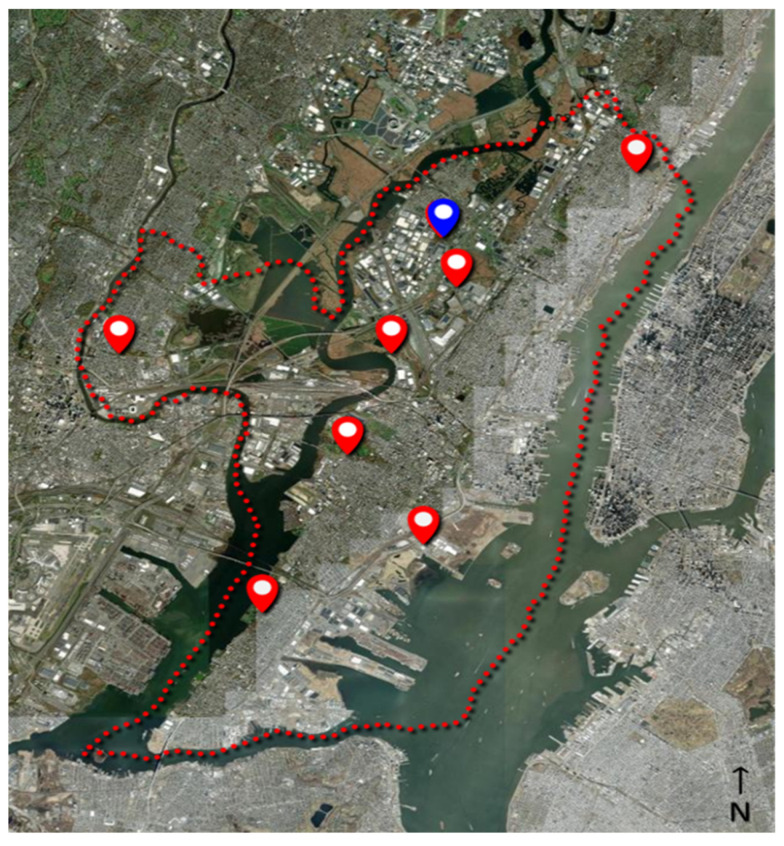
Location of physical traps (red markers) in Hudson County, NJ (red dotted line); the location of the eBoss instrument is indicated by the blue marker.

As part of an ongoing surveillance program, the Hudson Regional Health Commission maintains a series of eight light traps (Hausherr’s Machine Works, Toms River, NJ, USA) around Hudson County (NJ, USA) to monitor adult mosquito populations, see Figure 4. The traps utilize a clear 60 W incandescent light bulb to attract flying mosquitoes [40]. As the mosquitoes fly near the light, the downdraft from an electric fan forces the mosquitoes down a screen funnel into a collection jar. A dichlorvos-impregnated strip (AMVAC, Los Angeles, CA, USA) in the jar kills the mosquitoes to prevent escape. The trap is controlled by a photocell that runs the trap nightly from dusk to dawn throughout the mosquito season (May through October). Specimens are collected from the traps twice per week and delivered to the laboratory, where they are then counted and identified to species level under magnification utilizing a dichotomous key [41] by a certified mosquito identification specialist. The average trap count per day is calculated by dividing the number of captured mosquitoes by the number of days between trap collections and then averaged between all traps. The traps are placed around the county in the vicinity of known mosquito habitats (i.e., wetlands, coastal marshes, flood zones, etc.) in consideration of providing good dispersion across the entire county. Trapping sites are consistent each season and date back several decades. The average distance from the eBoss to the traps was 7.81 ± 4.6 km (range: 0.42–15.68 km).

## 3. Results

**Figure 5:**
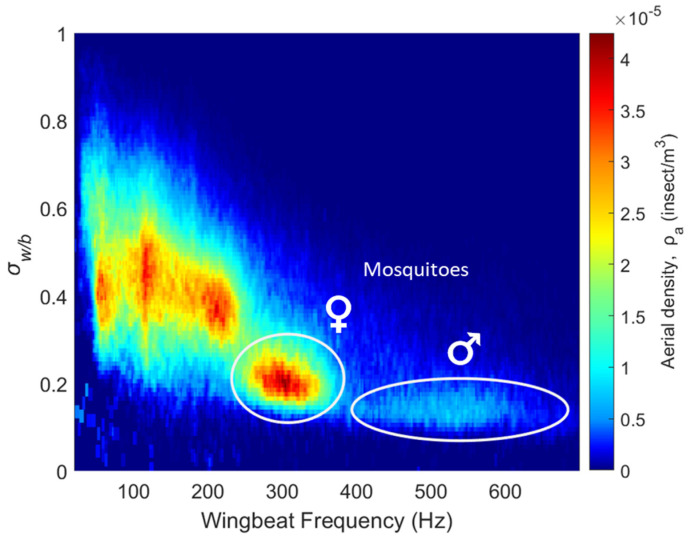
Distribution of different insect clusters showing aerial density; each bin is defined by optical extinction cross-section ratio and wingbeat frequency.

During the 8-month campaign, a total of 302,094 insect signals were recorded by the eBoss, while the total number of mosquitoes captured across all traps in 2022 was 6243. The case of multiple insects transiting through the beam at the same time is statistically rare, happening approximately once every 800 events; in these rare occurrences, the signal is treated like a regular event and only one event is recorded while the others are ignored. For each insect signal recorded by the optical sensor, the wingbeat frequency $f_{w}$ as well as the wing and body optical cross-sections $\sigma_{w}$ and $\sigma_{B}$ are retrieved, together with metadata (date and time of transit, transit duration, temperature, relative humidity, wind speed and direction, rainfall and UV and visible illuminance). Figure 5 presents the distribution of all insect signals as a function of the retrieved wingbeat frequency as well as the wing-to-body ratio $\sigma_{w/b}$. Due to the great diversity of flying insects in terms of body and wing size as well as wingbeat frequency, multiple clusters of insects are visible.

The top left part of the figure shows insects with large wings compared to their body and low wingbeat frequencies, typically Lepidoptera (such as butterflies and moths) and Odonata (dragonflies and damselflies). A large number of insect species share the region between 80 Hz and 250 Hz with wing-to-body ratios between 0.25 and 0.65; these clusters include many species of Diptera (flies), Hymenoptera (bees and wasps), Coleoptera (beetles), Orthoptera (grasshoppers and crickets) and Neuroptera (lacewings).

Species of flying insects with wingbeat frequencies above 250 Hz are scarcer. Mosquitoes are known to exhibit high wingbeat frequencies [42,43,44,45,46], with most species having female frequencies between 250 and 400 Hz and male frequencies between 350 and 700 Hz. This distinguishable high wingbeat frequency is particularly relevant in this study as it allows for the identification of the female and male mosquito clusters from their wingbeat frequency as well as their low wing-to-body ratios, marked by two white circles in Figure 5. These comprise 50,943, or 17%, of the total detections. Some small midges can potentially have wingbeat frequencies above 250 Hz; however, their wing optical cross-sections are below the detection limit of the instrument and their contribution would effectively not be observed in Figure 5.

To further confirm that these clusters are indeed female and male mosquitoes, a laboratory version of the eBoss measured transit signals from mosquitoes with the objective of comparing the wingbeat frequencies and wing-to-body ratios measured in laboratory conditions and in the field. A total of 45,777 transit signals were measured from five mosquito species: *Aedes aegypti*, *Aedes albopictus*, *Culex quinquefasciatus*, *Culex pipiens* and *Anopheles quadrimaculatus*. Insects were introduced to an enclosure positioned between the emitter and receiver of the eBoss. Table 1 presents the average retrieved wingbeat frequency and wing-to-body ratio as well as their respective standard deviations for both males and females in the laboratory and in the field.

While the mix of mosquito species studied in the laboratory is different from the one found in this field experiment, this is a further indication that those clusters are consistent with the presence of mosquitoes.

**Figure 6:**
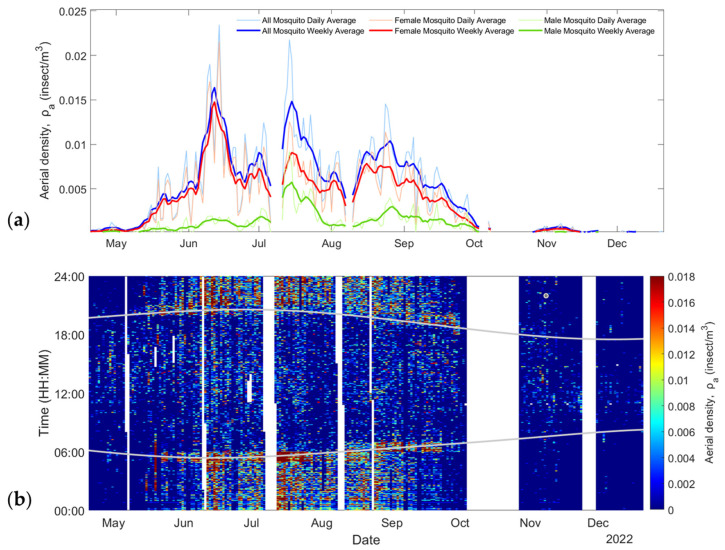
(**a**) Change in mosquito abundance throughout the entire season. (**b**) Mosquito aerial density as a date and time of the day; grey lines indicate sunrise and sunset times (EST, UTC-05:00).

Figure 6a shows the retrieved aerial density of male (green lines) and female (red lines) mosquitoes as well as the sum of both (blue lines) over the 8-month period. Figure 6b shows the aerial density for both males and females with a 1 min resolution. The grey lines display the sunrise and sunset times over the season, showing peaks of activity from mosquitoes at those times, which is coherent with the known behavior of mosquitoes [47].

**Figure 7:**
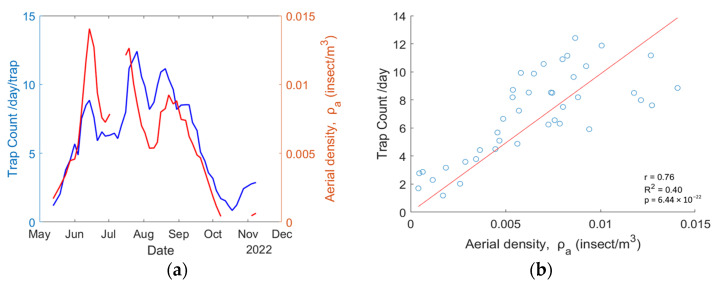
(**a**) Mosquito abundance data from the physical trap (blue line) and eBoss instrument (red line); (**b**) a scatterplot showing the correlation between trap count per day and aerial density.

The retrieved aerial density for mosquitoes is compared with the average trap count per day obtained by the Mosquito Program of the Hudson Regional Health Commission. Because both systems do not have the same temporal resolution, the aerial density from the eBoss is set to match the trap collection dates so both systems have artificially the same temporal resolution. The results are displayed in Figure 7a. Figure 7b presents the scatter plot for each point, showing a correlation of r = 0.76, $R^{2}$ = 0.40, and the *p* value is ${6.44\times 10}^{-22}$.

## 4. Discussion and Conclusions

The eBoss’s ability to run continuously, combined with the automatic detection of mosquitoes that eliminates manpower constraints, means that it can potentially achieve a much higher sampling rate than traditional methods. In this experiment, a single eBoss recorded 50,943 mosquito signals while the network of traps captured on average 780 captured insects per trap (6243 mosquitoes total). The total price of all traps used in the experiment is approximately USD 4000 total, and the operational cost to install them, collect captured insects and identify them was about USD 25,000 during the 2022 season, leading to roughly 4 USD/mosquito once the traps are purchased. By comparison, the eBoss is more expensive to build with a price in equipment alone estimated at around USD 3000 per unit (not including any construction labor). However, thanks to its automated data analysis, operational costs are low, assuming a 2 h maintenance every two weeks over the season; this leads to roughly 0.03 USD/mosquito during this field campaign.

**Figure 8:**
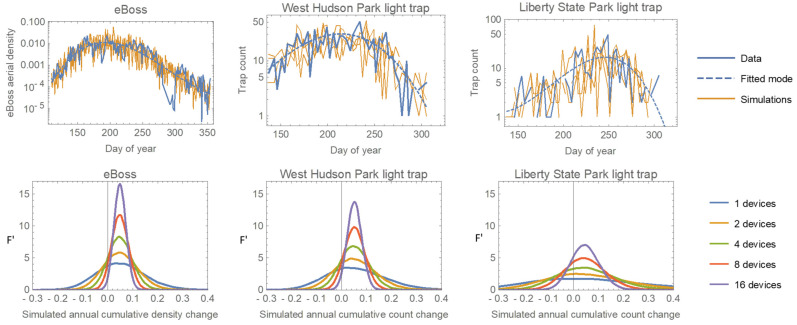
Top row: data collected by the eBoss and two light traps, the fitted within-year abundance trends, and two example simulations per device. Bottom row: The distributions of calculated proportional annual changes from pairs of simulations with the second simulation having a 5% increase added to the model (F’ is the normalized frequency density). The dotted vertical line shows the ‘true’ value of 0.05. The multiple curves for each device represent the combining of data from different numbers of devices.

The higher sample rate should translate into greater statistical power to detect both short-term changes and longer-term trends in mosquito abundance. To test this, we simulated data from both the light traps and the eBoss. We modeled the within-year temporal pattern of mosquito abundance as an ‘exponential cubic’ function $e^{\beta_{0}+\beta_{1}{t+\beta }_{2}t^{2}}$ where *t* is the ‘day of year’. For the trap data (counts), we directly fit a Negative Binomial regression. For the eBoss data (aerial densities), we fit a regular cubic regression to the log-transformed density data (log-transformation produced homoscedastic errors). We then simulated multiple years of data using the fitted models and error distributions, as well as models in which the cubic function was multiplied by 1.05 to represent a 5% increase in overall mosquito numbers. Figure 8 (top row) shows the original data, fitted model, and two simulated datasets, for the eBoss and two of the light traps.

As a simple test of relative power, we randomly selected 1, 2, 4, 8, or 16 simulations (representing hypothetical different numbers of deployed devices) from both the original and ‘5% increase’ models and calculated the percentage difference in their total annual counts/densities. We then repeated this for 10,000 comparisons at each device count. Each set of simulations produces a distribution of differences centered around the true value of 0.05, and the more devices, the narrower that distribution will be, indicating greater precision. Figure 8 (bottom row) shows that the eBoss has greater precision than the best trap (West Hudson Park) and much greater precision than one of the less effective traps. The comparison of curves from different numbers of devices showed that the eBoss has the same precision as 1.5 of the best traps and 6 of the worst. One caveat is that even the closest trap to the eBoss is far enough away that we cannot be sure they were sampling the same local aerial population of mosquitoes, so it is possible (though unlikely) that the eBoss’s excellent sampling performance is due to lucky placement in an area of high mosquito density.

This study provides promising indications that optical sensors, such as the eBoss system, can serve as valuable complementary tools to traditional trapping methods for monitoring mosquito abundance. The correlation observed between the abundance measurements from the eBoss and the physical traps suggests that optical sensors can produce reliable data. While traditional traps remain essential for their taxonomic accuracy and historical relevance, the eBoss system offers several additional benefits, including continuous, real-time monitoring and higher temporal resolution and sample size. These features enable the detailed tracking of mosquito activity patterns, which can enhance the effectiveness of surveillance efforts. The results of this field campaign highlight the potential for integrating optical sensors into existing mosquito monitoring programs. By complementing trap data with high-resolution, automatically collected information from systems like eBoss, vector control programs can gain more comprehensive insights into mosquito population dynamics.

Future studies should aim to further validate these findings by co-locating multiple eBoss devices with traps placed directly alongside the beam. Such research will help to refine the technology, improve its accuracy and ensure its effectiveness and reliability, paving the way for broader application in public health surveillance. Finally, additional work is underway to improve the taxonomic resolutions of optical sensors so that the abundance of individual species can be evaluated. Being a recent technology, we believe that further laboratory work may significantly improve the taxonomic resolution of entomological optical sensors.

## Figures and Tables

**Table 1:** Wingbeat frequency and wing-to-body ratio data for male and female mosquitoes.

	Field Data	Laboratory Data
Male wingbeat frequency	524 ± 65 Hz	534 ± 176 Hz
Male wing-to-body ratio	0.19 ± 0.05	0.11 ± 0.08
Female wingbeat frequency	300 ± 30 Hz	345 ± 47 Hz
Female wing-to-body ratio	0.27 ± 0.07	0.21 ± 0.15

## Data Availability

The data presented in this study are available on request from the corresponding author.
